# Five risk factors and their interactions of probability for a sow in breeding herds having a piglet death during days 0–1, 2–8 and 9–28 days of lactation

**DOI:** 10.1186/s40813-021-00231-0

**Published:** 2021-08-30

**Authors:** Yuzo Koketsu, Ryosuke Iida, Carlos Piñeiro

**Affiliations:** 1 School of Agriculture, Meiji University, Higashi-mita 1-1-1, Tama-ku, Kawasaki, Kanagawa 214-8571 Japan; 2 PigCHAMP Pro Europa S.L., Calle Dámaso Alonso, 14, 40006 Segovia, Spain

**Keywords:** Farm data, Piglet death, Pre-weaning mortality, Sow data

## Abstract

**Background:**

Increasing preweaning piglet mortality is a concern for veterinarians and producers in relation to sow performance and piglet welfare. Our objectives were (1) to characterize pre-weaning piglet mortality risk for sows (PWM) during early (0–1 days), mid- (2–8 days) and late (9–28 days) lactation and (2) to quantify the following five factors and their interactions, parity, number of piglets born alive (PBA), number of stillborn piglets (SB), gestation length (GL) and season for PWM during the three lactation phases.

**Methods:**

Data obtained from 264,333 parity records of 55,635 sows farrowed in 2015 and 2016 from 74 Spanish herds. Three multi-level mixed-effects logistic regression models were separately applied for PWM during three lactation phases, which was analyzed as whether or not a sow had a piglet death (i.e. probability of a sow having a piglet death) in each phase.

**Results:**

PWM during early, mid- and late lactation were 36.9, 27.0 and 15.4%, respectively. As PBA increased from 11 or less to 16 or more pigs, PWM during early and mid-lactation increased by 15.8 and 6.0%, respectively, but there was no increase during late lactation. Also, as GL decreased from 117–120 to 110–113 days, PWM during early, mid- and late lactation increased by 7.5, 6.8 and 1.5%, respectively. Additionally, PWM during the respective lactation phases increased by 8.3, 5.2 and 1.0%, as SB increased from 0 to 3 or more pigs. During early lactation, parity 1 sows had 2.1% lower PWM than parity 5 or higher sows, but during mid- and late lactation they had 4.2% higher PWM (*P* < 0.05). However, there was no difference between summer and winter for PWM during early lactation (*P* = 0.26).

**Conclusion:**

Management practices to reduce PWM need to take account of these factors, and be modified for different phases. For example, during early lactation special care should be given to piglets born to parity 5 or higher sows farrowing 16 or more PBA, having 3 or more SB or GL 110–113 days, whereas during mid- and late lactation more care should be given to piglets born to parity 1 sows with the same PBA, GL and SB conditions.

**Supplementary Information:**

The online version contains supplementary material available at 10.1186/s40813-021-00231-0.

## Introduction

An increased number of piglet deaths during lactation is one of the biggest concerns of vets and producers related to sow performance and piglet welfare [1–3]. The typical way of monitoring piglet deaths during lactation has been to record them as herd-level preweaning piglet mortality, which is calculated by the recording systems [4]. However, if piglet deaths are recorded as herd-level information, it is not possible to perform multivariable analyses to examine multiple factors and their interactions in the same model [5, 6].

However, there are several software products that record piglet deaths and can generate data of sow-level piglet deaths during different pre-weaning stages during lactation. Using such a data, possible factors for pre-weaning piglet mortality risk for sows (PWM) as probabilities of a sow having a piglet death could be separately examined during each of three lactational phases. Also, our idea is to analyze PWM as a binary outcome during early, mid- and late lactation, because unlike sow deaths, piglet death events can occur multiple times at the sow level, with several piglets in a litter possibly dying at different times during a single lactation. A binary model has been applied for piglet deaths or stillbirths because those are a zero-inflated variable [6–8]. Therefore, it is now possible to conduct sow-level PWM during different lactation phases, but to date no such studies have been reported.

Possible factors associated with higher PWM are parity, more piglets born alive (PBA), more stillborn piglets (SB), shorter gestation length (GL) and farrowing season [1, 2, 9]. Various studies have found links between these factors and issues that could be associated with pre-weaning piglet deaths. For example, low and high parity have been associated with higher pre-weaning mortality [9–11]. Also, more SB at herd-level has been correlated with herd-level pre-weaning mortality and post-weaning mortality [12]. Furthermore, sows with shorter GL have been associated with having more SB and more PBA which likely results in lower litter weaning weights [13] which in turn would be related to piglet deaths. However, there have been conflicting results about associations between farrowing season and piglet deaths [1, 2]. For example, researchers suggested that pre-weaning piglet mortality was higher in winter, due to cold stress, than in the other seasons [1], whereas another report observed higher preweaning piglet mortality in summer than in spring due to heat stress [14].

No research has studied these five factors (i.e. parity, PBA, SB, GL and season) and their interactions for PWM during early, mid- and late lactation in the separate three models. Therefore, our objectives were (1) to characterize PWM during early (0–1 days), mid- (2–8 days) and late (9–28 days) lactation in sow herds, and (2) to quantify the five factors and their two-way interactions for PWM during these three phases of lactation.

## Materials and methods

### Farms and sow measurement records with ages at piglet death

A veterinary clinic (PigCHAMP Pro Europa S.L. Segovia, Spain) has requested all client producers to mail their data files if they consented to allow their data to be used for research purposes under their data-share program, and has accumulated a database. The data in the present project were extracted from the sow database**.** Therefore, the present study was designed as an observational study coordinating sow data from 91 Spanish herds which had 10-year records, and their farm data had been previously used for a herd-level longitudinal study [15]. For this project, we used two years of sow data from 2015 to 2016.

However, not all of the herds could be used in the analysis because not all producers appeared to have properly recorded the ages of the piglets that died. Consequently, 17 of the herds were removed because their records had no piglet deaths during days 0–1 of lactation throughout the 2 studied years and it was considered that those producers had not properly recorded the ages at piglets died. The 74 remaining herds had records of piglet deaths in all three lactation phases. Also, according the EU regulation [16], sow records with lactation length 20 days or fewer were not used for the present study. Therefore, the initial sow data contained 276,260 parity records of 59,088 sows farrowed in 74 herds between 2015 and 2016. Mean herd size in the 74 herds over the two years was 1,066 (874 standard deviation) sows ranging from 83 to 3,682 sows.

### Data and exclusion criteria

Parity records in the data were excluded if they met any of the following criteria: total number of piglets born being 0 or 31 pigs or more (181 records) [17]; lactation length of 41 or more days (3095 records), nurse sow records (7179 records) and GL of 109 days or less or 121 days or more (1472 records). The range of gestation length was chosen as 115 ± 5 days. Also, the nurse records were not used because there were few records, and they were not evenly distributed across all the farms. For example, 15 farms had only 1 or 0 nurse records during the studied years. Therefore, the final data contained 264,333 parity records of 55,635 sows.

Regarding the ages of piglet deaths recorded by the produce, sow records were excluded if the average age of piglet deaths was the same or one day less than the average weaning age (7729 records). These data were excluded because it was considered that in these records the dates of piglet death events had been recorded at weaning or one day before weaning, and did not show the actual piglet death date. Therefore, the final data for piglet ages contained 256,604 records.

The recording accuracy of piglet death events may vary between farms. So, to check how accurately the producers had recorded piglet death events, herd-level internal consistency was examined by comparing the pre-weaning mortality recorded by the producers over the two studied years with that calculated by the software. The comparison showed that the producers recorded 85.3% of the pre-weaning mortality calculated by the software, i.e. that they had recorded 85.3% of the piglet deaths occurring during lactation. The Pearson correlation coefficient between the mean pre-weaning mortality values calculated by the two methods in the 74 herds over the two years was 0.74 (*P* < 0.01).

### Definitions and categories

Sows were categorized into three parity groups: 1, 2–4 and 5 or higher. There were four farrowing season groups: January-March (winter); April-June (spring), July–September (summer) and October–December (autumn). Three BA groups were based on the 25th and 75th percentiles of BA: 11 pigs or fewer, 12–15 pigs and 16 pigs or more. Three GL groups were 110–113, 114–116 and 117–120 days. The four SB groups were 0, 1, 2 and 3 or more pigs.

### Statistical analysis

All analyses were carried out using of SAS University Edition (SAS Inst. Inc., Cary, NC, U.S.A.). Also, a piglet death was treated as a binary outcome and PWM was examined as the probability of a sow having a piglet death. For the binary outcome, a three-level generalized mixed-effects model was used with a logit link function in individual parity records for each phase of lactation. This model was used to account for the clustering of sows within a farm (GLIMMIX, random statement), and the correlation between repeated measures in the same sow within a farm (GLIMMIX, random _residual_ statement). The ILINK (inverse link function) was used to convert the logarithm to a probability [18]. The models contained the five factors, parity, BA, SB, GL and season, and 10 possible two-way interactions as fixed effects. Additionally, farrowing year and mean herd size (sows) for the two studied years were included as fixed effects in all models. All significance levels were set at *P* < 0.05. Also, pairwise multiple comparisons were performed using the Tukey–Kramer test when significance was found. The adequacy of the model assumptions for the random effects was checked by visual inspection of normal-probability plots [19]. Additionally, Spearman correlation analysis was performed between the three lactation phases for piglet deaths.

### Intraclass correlation coefficient

To evaluate the variation in the amount of PWM in the three lactation phases that could be explained by the farm or sow, the intraclass correlation coefficients (ICC) were calculated by the following equations [20]:

ICC (individual records within the same farm but different sows) = ${\sigma }_{v}^{2}/({\sigma }_{v}^{2}+{\pi }^{2}/3)$,

ICC (individual records within the same farm) = $({\sigma }_{v}^{2}+{\sigma }_{u}^{2})/({\sigma }_{v}^{2}+{\sigma }_{u}^{2}+{\pi }^{2}/3)$,

in which ${\sigma }_{v}^{2}$ is the between-farm variance,${\sigma }_{u}^{2}$ is between-sow variance at the individual record level and ${\pi }^{2}/3$ is the assumed variance at the individual record level.

## Results

**Fig. 1:**
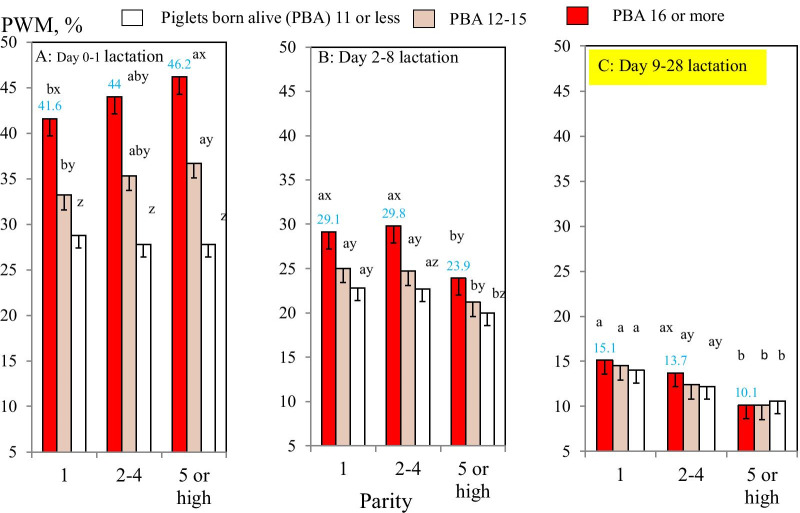
**A**–**C** Two-way comparisons of pre-weaning piglet mortality risk for sows (probabilityof a sow having a piglet death: PWM) during early (0–1 days), mid-(2–8 days) and late (9-28 days) lactation (N = 264,333 sows) between parity and PBA groups. Means and SEs were estimated in mixed-effects models. ^a, b^Different superscripts within parity groups represent significant differences in means (*P* < 0.05). ^x–z^Different superscripts within PBA groups represent significant differences in means (*P* < 0.05)

**Fig. 2:**
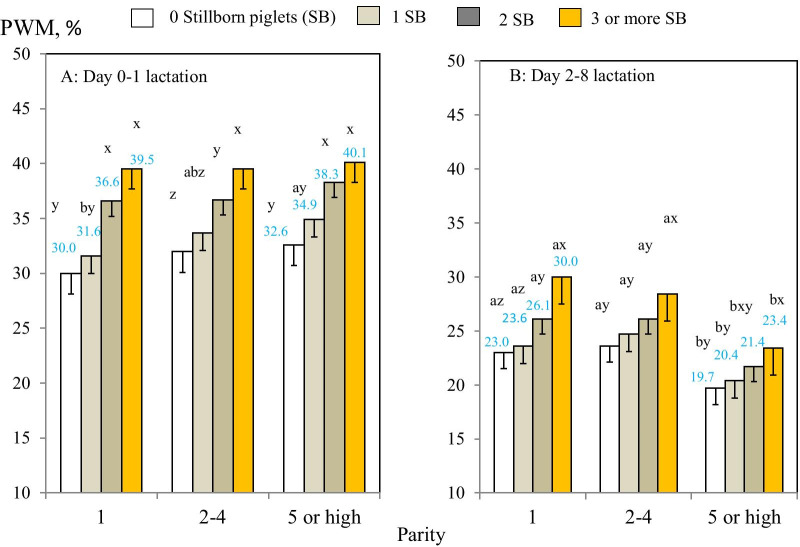
**A**, **B** Two-way comparisons of pre-weaning piglet mortality risk for sows (probabilityof a sow having a piglet death: PWM) during early (0–1 days) and mid-(2–8 days) lactation (N = 264,333 sows) between parity and SB groups. Means and SEs were estimated in mixed-effects models. ^a–c^Different superscripts within parity groups represent significant differences in means (*P* < 0.05). ^x,y^Different superscripts within SB groups represent significant differences in means (*P* < 0.05)

**Fig. 3:**
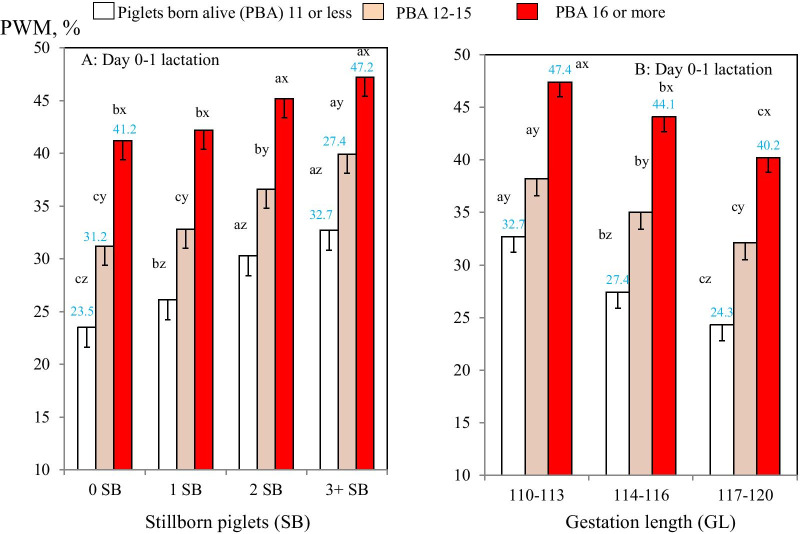
AB. Two-way comparisons of pre-weaning piglet mortality risk for sows (probabilityof a sow having a piglet death: PWM) during early (0–1 days) lactation (N = 264,333 sows) between PBA groups and either SB (**A**) or gestation length (**B**) groups. Means and SEs were estimated in mixed-effects models. ^a–d^Different superscripts within SB or GL groups represent significant differences in means (*P* < 0.05). ^x–z^Different superscripts within PBA groups represent significant differences in means (*P* < 0.05)

**Fig. 4:**
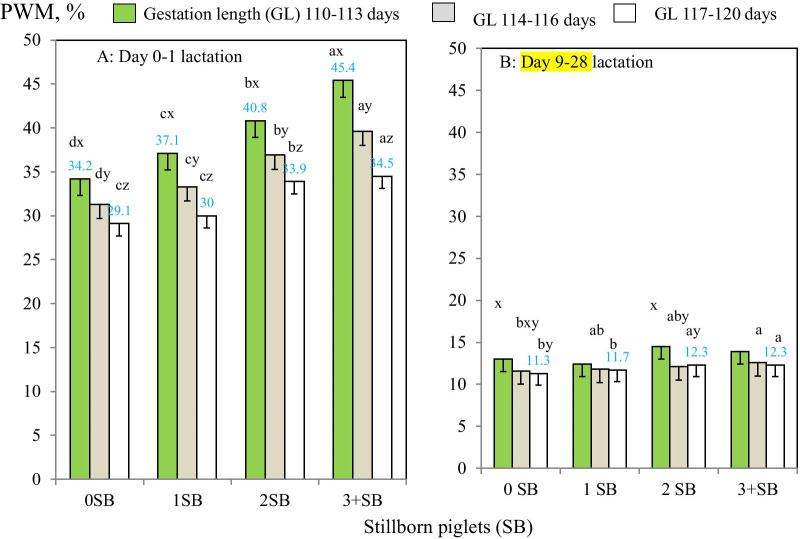
AB. Two-way comparisons of pre-weaning piglet mortality risk for sows (probabilityof a sow having a piglet death: PWM) during early (0–1 days) and late lactation (9–28 days; N = 264,333 sows) between gestation length groups and either parity (**A**) or SB groups (**B**). Means and SEs were estimated in mixed-effects models. ^x–z^Different superscripts within SB groups represent significant differences in means (*P* < 0.05). ^a–d^Different superscripts within gestation length groups represent significant differences in means (*P* < 0.05)

Mean PWM during early, mid- and late lactation were 36.9, 27.0 and 15.4%, respectively (Table 1). Table 2 shows P-values of the five factors and their two-way interactions in the models for PWM during each of the three lactation phases. All the five factors: parity, season, PBA, SB and GL were significant for PWM during early and mid-lactation (*P* < 0.05; Table 2). During late lactation, parity, SB and GL were significant for PWM (*P* < 0.01), but season and PBA were not (*P* ≥ 0.08). There were also some significant interactions for PWM between the five factors, such as parity × PBA, parity × SB, PBA × GL, PBA × SB and SB × GL (Table 2; Figs. 1, 2, 3 and 4). Additional file 1 shows estimates of fixed effects and random effect variance included in the three models for PWM. Also, 34–45% of ICCs within the same sow and within the same farm were found in the two models for mid- and late lactation (Additional file 1).

**Table 1:** Summary statistics for piglet deaths and related measurements

Measurements	N	Mean (± SD)	Minimum	Maximum
Farrowed parity	264,333	3.6 (2.1)	1	17
Gestation length, days	264,333	115 (1.5)	110	120
Number of piglets born alive	264,333	13.4 (3.5)	1	29
Number of stillborn piglets	264,333	1.06 (1.50)	0	23
Lactation length, days	264,333	25.1 (3.4)	21	40
Number of piglets weaned	264,333	11.5 (2.2)	0	39
Age when piglet died, days	256,604	2.40 (4.52)	0	38
Probability of a sow having a piglet death during early lactation (days 0–1), %	256,604	36.9 (48.2)	–	–
Probability of a sow having a piglet death during mid-lactation (days 2–8), %	256,604	27.0 (44.3)	–	–
Probability of a sow having a piglet death during late lactation (days 9–28), %	256,604	15.4 (36.1)	–	–

*SD* standard deviation

**Table 2:** *P*-values of fixed factors included in the mixed-effects logistic regression models for pre-weaning piglet mortality risk for sows (probabilities of a sow having a piglet death) during early (0–1 days), mid- (2–8 days) and late (9–28 days) lactation

Fixed factors^1,2^	Pre-weaning piglet mortality risk		
	Early	Mid	Late
	*P*-value	*P*-value	*P*-value
Parity groups	0.04	< 0.01	< 0.01
Season groups	0.02	0.01	0.10
Piglets born alive (PBA)	< 0.01	< 0.01	0.15
Stillborn piglets (SB)	< 0.01	< 0.01	< 0.01
Gestation length (GL)	< 0.01	< 0.01	< 0.01
*Two-way interactions*			
Parity × season	0.63	0.42	0.05
Parity × PBA	< 0.01	0.04	< 0.01
Parity × GL	0.13	0.27	0.16
Parity × SB	0.02	0.03	0.23
Season × PBA	0.20	0.20	0.05
Season × GL	0.98	0.54	0.37
Season × SB	0.33	0.45	0.08
PBA × GL	< 0.01	0.28	0.44
PBA × SB	< 0.01	0.06	0.09
SB × GL	< 0.01	0.05	0.03

Table 3 shows comparisons for PWM between the groups for each of the five factors during each lactation phase. With regard to PBA, during early lactation, PWM increased from 28.1% in the PBA 11 or less pigs group to 43.9% in the PBA 16 or more pigs group (i.e. an increase of 15.8%). During mid-lactation, the increase was from 21.5 to 27.5%, but there was no increase during late lactation (*P* = 0.15). Also, with regard to SB, during early lactation PWM increased from 31.5% in sows with SB 0 to 39.8% in sows with SB 3 or more pigs, and from 22.0 to 27.2% during mid-lactation and from 12.0 to 12.9% during late lactation, respectively. Additionally, with regard to GL, during early lactation PWM increased from 31.8% for GL 117–120 days to 39.3% for GL 110–113 days, and from 21.4 to 28.2% during mid-lactation and from 11.9 to 13.4% during late lactation, respectively.

**Table 3:** Comparisons of pre-weaning piglet mortality risk for sows (probabilities of a sow having a piglet death) during early (0–1 days), mid- (2–8 days) and late (9–28 days) lactation between parity, farrowing season, piglets born alive, gestation length and stillborn piglets groups^1^

Groups	N	Pre-weaning piglet mortality risk		
		Early lactation	Mid-lactation	Late lactation
		Mean (± SE)	Mean (± SE)	Mean (± SE)
Parity				
1	55,635	34.3 (1.60)^b^	25.5 (2.53)^a^	14.5 (1.91)^a^
2–4	123,314	35.4 (1.72)^ab^	25.7 (2.34)^a^	12.7 (1.74)^b^
5 or higher	85,384	36.4 (1.74)^a^	21.3 (2.20)^b^	10.3 (1.45)^c^
Farrowing season				
Jan.–Mar	64,584	35.5 (1.97) ^ab^	22.3 (2.36)^b^	12.1 (1.72)
Apr.–Jun	64,264	35.2 (1.58) ^ab^	24.0 (2.40)^ab^	12.4 (1.71)
Jul.–Sept	68,990	34.0 (1.54) ^b^	25.5 (2.40)^a^	13.3 (1.84)
Oct.–Dec	66,495	36.8 (1.88) ^a^	24.7 (2.32)^a^	11.9 (1.54)
Piglets born alive				
11 or less pigs	69,232	28.1 (1.52)^c^	21.5 (2.14)^c^	12.2 (1.65)
12–15 pigs	95,371	35.0 (1.61)^b^	23.6 (2.21)^b^	12.3 (1.63)
16 or more pigs	99,730	43.9 (1.92)^a^	27.5 (2.80)^a^	12.8 (1.71)
Stillborn piglets				
0 pigs	127,524	31.5 (1.48)^d^	22.0 (2.09)^d^	12.0 (1.59)^b^
1 pig	65,185	33.0 (1.62)^c^	22.8 (2.20)^c^	11.9 (1.60)^b^
2 pigs	38,494	37.2 (1.73)^b^	24.5 (2.36)^b^	12.9 (1.71)^a^
3 or more pigs	33,130	39.8 (1.98)^a^	27.2 (2.60)^a^	12.9 (1.74)^a^
Gestation length, days				
110–113 days	37,877	39.3 (1.93)^a^	28.2 (2.63)^a^	13.4 (1.81)^a^
114–116 days	187,339	35.2 (1.68)^b^	23.1 (2.24)^b^	12.0 (1.61)^b^
117–120 days	39,117	31.8 (1.49)^c^	21.4 (2.06)^c^	11.9 (1.57)^b^

^1^Means and SEs were estimated in mixed-effects models

^a^^−^^c^ Different superscripts within a column represent significant differences in means (*P* < 0.05)

The association between parity and PWM varied during lactation phases. For example, as the number of parity increased, PWM during early lactation increased from 34.3 to 36.4%, whereas during mid- and late lactation PWM decreased from 25.5 to 21.3% and from 14.5 to 10.3%, respectively (Table 3; *P* < 0.05). In season groups, sows farrowed in summer had higher PWM during mid-lactation than in winter (Table 3; *P* < 0.05), but there was no difference between summer and winter for PWM during early lactation (*P* = 0.26). Furthermore, no significant differences were found between farrowing season groups for PWM during late lactation (*P* = 0.10). Additionally, there were no two-way interactions between farrowing season and either parity, PBA, SB or GL groups for PWM during any lactation phase (Table 2; *P* > 0.05).

There were significant two-way interactions between parity and PBA groups for PWM during early, mid- and late lactation (*P* < 0.05; Table 2; Fig. 1A–C; Additional file 2). For example, in the PBA 16 or more group, as parity increased from parity 1 to parity 5 or higher, PWM during early lactation increased from 41.6 to 46.2% (Fig. 1A). However, in the same PBA group (16 or more), PWM during mid-lactation decreased from 29.1% in parity 1 to 23.9% in parity 5 or higher (Fig. 1B). Also, PWM during late lactation decreased from 15.1 to 10.1% in the PBA 16 or more group (Fig. 1C).

There were also significant two-way interactions between parity and SB groups for PWM during early and mid-lactation (*P* < 0.05; Table 2; Fig. 2AB; Additional file 3). For example, for parity 1 sows PWM during early lactation increased from 30.0% for sows with SB 0 to 39.5% for sows with SB 3 or more, whereas for parity 5 or higher sows, PWM in the respective SB groups increased from 32.6% and 40.1% (Fig. 2A). Also, during mid-lactation, PWM in parity 1 sows increased from 23.0% for sows with SB 0 to 30.0% for sows with SB 3 or more, whereas for parity 5 sows the increase in PWM between the SB 0 and 3 or more groups was only from 19.7 to 23.4% (*P* < 0.05; Fig. 2B).

Two-way interactions between PBA and both SB groups and GL groups were significant for PWM only during early lactation (*P* < 0.05; Table 2; Fig. 3A, B; Additional file 4). Firstly, for sows with SB 3 or more, PWM increased from 32.7% in sows with PBA 11 or less to 47.2% for sows with PBA 16 or more, whereas for sows with SB 0 PWM increased from 23.5 to 41.2% in the respective PBA groups (Fig. 3A). Also, for the PBA 16 or more group, PWM increased from 40.2% in the GL 117–120 group to 47.4% in the GL 110–113 group, whereas for sows with PBA 11 or less, PWM increased from 24.3 to 32.7% in those 3 GL groups (Fig. 3B).

There were also significant two-way interactions between GL groups and SB groups for PWM during early and late lactation (*P* < 0.05; Table 2; Fig. 4AB; additional file 5). During early lactation, when GL was 110–113 days, PWM increased from 34.2% for sows with SB 0 to 45.4% for sows with SB 3 or more, whereas when GL was 117–120 days, PWM increased from only 29.1 to 34.5% in the four SB groups (Fig. 4A). However, during late lactation while PWM for sows with GL 117–120 days increased from 11.3% for sows with SB 0 to 12.3% for sows with SB 3 or more, there was no difference in PWM for sows with SB 3 or more in the different GL groups (*P* > 0.28; Fig. 4B).

In correlation analysis, significant positive coefficients were found (*P* < 0.01; Additional file 6) for the numbers of piglet deaths between early and mid-lactation (Coeffcient: 0.02), as well as between those during early and late lactation (Coeffcient: 0.02) and between mid- and late lactation (Coefficient: 0.12).

## Discussion

This is the first study about how these five factors (i.e. parity, season, PBA, SB and GL) and their interactions have different associations with PWM during the three different lactation phases. Also, the impact of the five factors on PWM differed between lactation phases. Therefore, to reduce piglet deaths, it is recommended that at each of the three lactation periods producers with veterinarians prioritize care provision for high-risk sows, such as high and low parity sows that farrow more PBA or have more SB or with short GL.

Our study also suggests that the impact of PBA on PWM is much larger during early lactation than during late lactation (15.8% vs. no increase). Also, this large impact of increased PBA during early lactation meant that it had a larger impact on PWM than any of the other four factors assessed in our study; 15.8% increased PWM due to increased PBA, compared with only 2.1% due to increased parity number, 7.5% due to decreased GL, 8.3% due to increased SB, and only 2.8% between summer and autumn.

Our analysis showed a substantial association between PWM and SB 3 or more. Other studies have also found associations between increased SB and sow performance and piglet welfare, such as decreased litter weights at weaning [13], more occurrences of uterine prolapse [21] or more abortions at subsequent pregnancy [22], decreased farrowing rate, and decreased PBA at subsequent parity [13]. Also, SB data appears to contain piglets that died immediately after farrowing [23]. So, these associations suggest that sows with increased SB may have had farrowing difficulty [1, 2] or an infectious disease problem such as porcine parvovirus or porcine reproductive and respiratory syndrome virus [24]. Also, in order to decrease the number of births of weak piglets and the number of piglet deaths immediately after birth, assisted farrowing with timely use of manual delivery techniques should be refined with herd health programs [2, 9].

Our study also showed that parity 1 sows had higher PWM than parity 5 or higher sows during mid- and late lactation, compared with lower PWM during early lactation. A possible reason for the higher PWM for parity 1 sows during mid- and late lactation could be that sows produce less colostrum in parity 1 than in parity 2 or higher [25, 26]. Also, some management practices such as colostrum intake assistance or split nursing can help small piglets survive during early lactation [27–29], but these practices may not necessarily help to reduce piglet deaths during mid- or late lactation [15], especially for sows in parity 1 that are having their first experience of being suckled. Furthermore, parity 1 sows might be more susceptible to poor herd health situations because they have immune immaturity [2]. So, herd health programs with special care for parity 1 sows should be reconsidered to decrease PWM [1, 2, 24].

In contrast, the high PWM in high parity sows during early lactation could have been due to many PBA. A previous study reported that high parity sows farrowed many PBA with uneven size and low vitality, as well as having a long farrowing duration [30]. Also, it appears that high parity sows would have more matured immunity and more experiences for lactating than parity 1 sows. Therefore, our findings suggest that during early lactation special care and attention should be given to piglets born to party 5 or higher sows, whereas during mid- and late lactation more care should be given to piglets born to parity 1 sows.

Shorter GL is thought to be associated with higher estrogen concentrations and larger litters; sows with larger litters have a larger fetal placental unit which produces higher concentrations of estrogen near parturition, and increases the release of oxytocin and prostaglandin [31]. Our results suggested that pregnant pigs with many fetuses (to be many PBA) had shorter GL and that short GL caused SB to increase. All these effects resulted in higher PWM during all lactation phases. Therefore, pregnant pigs should be moved to a farrowing barn at least 6 days before their due date. Also, sows with GL 110 or 111 days might have had an abortion due to some diseases.

The two-way interactions in our study between PBA and either GL or SB for PWM during early lactation indicate that when sows farrowed PBA 16 or more and had either 3 or more SB or 110–113 days GL, there was an increase in PWM during early lactation. A possible reason for the increased PWM under these situations is that shorter GL and more SB could have increased the number of births of weak piglets which would be more likely to die during early lactation, especially when the sows were in parity 1.

Our study showing the high PWM during mid-lactation in summer agrees with a previous study showing the high pre-weaning mortality risks in summer in Japanese herds [14]. Also, during early lactation, there was no difference of PWM between summer and winter in our study. It appears that capabilities to cope with heat stress or winter coldness in different lactation phases vary between farms. However, with advanced facilities, equipment (e.g. cool cell or evaporating cooling systems) and management [9, 32], it is possible that producers are able to mitigate the seasonal effects on piglet deaths for sows fed in confined barns.

The various two-way interactions between four of the factors (i.e. PBA, parity, GL and SB) for PWM during the different lactation phases indicate that there are different relationships between the four factors depending on whether PWM occurred during early, mid- and late lactation. Previous studies have reported that higher PBA in higher parities increased SB [12, 33], leading to increased PWM especially during early lactation. Similarly, in our study the impact of having 16 or more PBA on PWM was greatest during early lactation. These results indicate that prioritized management practices are needed to decrease piglet deaths for sows that farrow many PBA during early lactation, such as supervised and assisted farrowing with heater management [9, 34, 35]. Also, it is critical to monitor and provide care to piglets born to other high risk sows (e.g. GL 110–113 days, SB 2 or more) to reduce piglet deaths in each of three lactation phases.

Also, compared to the low ICC (i.e. 4.0% within the same sow and 2.4% within the same farm) in a previous PBA model [36], there was a higher ICC for PWM during mid- and late lactation (i.e. 34–45%) in the present study. These high ICCs suggest that sows having high PWM during mid- and late lactation tend to have high PWM during the same lactation phase across parity, and farms having high PWM tend to have high PWM in sows. These findings suggest that producers should carefully monitor sows that had high piglet deaths in earlier records.

Finally, the modestly positive correlation between piglet deaths during mid-lactation and late lactation suggests that high PWM during mid-lactation can increase PWM during late lactation, but the low correlation coefficients between early and either mid- or late lactation indicate that sows having high PWM during early lactation do not necessarily continue to have high PWM during mid- or late lactation.

In conclusion, more PBA, more SB, shorter GL and both low and high parity were important factors for increased PWM, during all lactation phases, but the size of impact of each factor varied between the three lactation phases. Therefore, management practices to reduce piglet deaths need to take account of PBA, SB, GL and parity, and be adjusted depending on the phase of lactation.

There are some limitations in this observational study that should be mentioned. First, euthanized piglets were not recorded. Secondly, cross-fostered piglets might have increased PWM during mid- and late lactation. Additionally, our study herds differed in their genetic programs, location, herd health conditions, facilities and integrated production companies, and these differences were not taken into account in our analysis. Also, the accuracy of recordings done by the producers could vary between farms [37]. However, our three-level statistical models contained a random effect of the farm to explain some of the variation between the studied herds.

## Supplementary Information

Additional file 1. P-value and estimates of fixed factors and random effect variance included in the mixed-effects logistic regression models for pre-weaning piglet mortality risk for sows (probabilities of a sow having a piglet death) during early (0-1 days), mid- (2-8 days) and late (9-28 days) lactation.

Additional file 2. Two-way comparisons of pre-weaning piglet mortality risk for sows (probabilities of a sow having a piglet death: PWM) during early (0-1 days), mid- (2-8 days) or late (9-28 days) lactation between parity and piglets born alive (PBA) groups^1^.

Additional file 3. Two-way comparisons of pre-weaning piglet mortality risk for sows (probabilities of a sow having a piglet death: PWM) during early (0-1 days) or mid- (2-8 days) lactation between parity and stillborn piglet groups^1^.

Additional file 4. Two-way comparisons of pre-weaning piglet mortality risk for sows (probabilities of a sow having a piglet death: PWM) during early-lactation (0-1 days) between piglets born alive groups and gestation length groups, and between piglets born alive groups and stillborn piglet groups^1^.

Additional file 5. Two-way comparisons of pre-weaning piglet mortality risk for sows (probabilities of a sow having a piglet death: PWM) during early (0-1 days) or late (9-28 days) lactation between gestation length and stillborn piglet groups^1^.

Additional file 6. Spearman correlation matrix for the number of piglet deaths during early (0-1 days), mid- (2-8 days) or late (9-28 days) lactation.

## Data Availability

The dataset analyzed during the current study is not publicly available because producers’ privacy could be compromised.

## Abbreviations

PBA: Piglets born alive
GL: Gestation length
SB: Stillborn piglets
