# Supplementary material for: Five risk factors and their interactions of probability for a sow in breeding herds having a piglet death during days 0–1, 2–8 and 9–28 days of lactation
Source: Porcine Health Manag. 2021 Aug 30;7:50. doi: 10.1186/s40813-021-00231-0 (PMC8404260; doi:10.1186/s40813-021-00231-0)
Supplement: Supplementary file 1 — P-value and estimates of fixed factors and random effect variance included in the mixed-effects logistic regression models for pre-weaning piglet mortality risk for sows (probabilities of a sow having a piglet death) during early (0-1 days), mid- (2-8 days) and late (9-28 days) lactation. [file 40813_2021_231_MOESM1_ESM.docx]

**Additional file 1.** P-value and **e**stimates of fixed factors and random effect variance included in the mixed-effects logistic regression models for pre-weaning piglet mortality risk for sows (probabilities of a sow having a piglet death) during early (0-1 days), mid- (2-8 days) and late (9-28 days) lactation

| Fixed and | |  | | | | | |  | | | Lactation phase |  |  |  |  |
| --- | --- | --- | --- | --- | --- | --- | --- | --- | --- | --- | --- | --- | --- | --- | --- |
| random | | Early | | | | | |  | | | Mid |  | Late |  |  |
| effects^1,2^ | | Estimate (± SE) | | | | | | P-value | | | Estimate (± SE) | P-value | Estimate (± SE) | P-value | |
| Intercept | | -1.109 (0.204) | | | | | | < 0.01 | | | - 1.495 (0.248) | < 0.01 | -2.486 (0.247) | < 0.01 |  |
| Parity groups | |  | | | | | | 0.04 | | |  | < 0.01 |  | < 0.01 |  |
| 1 | | 0.090 (0.082) | | | | | |  | | | 0.337 (0.098) |  | 0.413 (0.116) |  |  |
| 2-4 | | 0.004 (0.056) | | | | | |  | | | 0.240 (0.081) |  | 0.242 (0.084) |  |  |
| Season groups | |  | | | | | | 0.02 | | |  | 0.01 |  | 0.10 |  |
| Jan.-Mar. | | - 0.080 (0.088) | | | | | |  | | | - 0.171 (0.098) |  | 0.075 (0.103) |  |  |
| Apr.-Jun. | | - 0.014 (0.077) | | | | | |  | | | - 0.026 (0.081) |  | 0.115 (0.096) |  |  |
| Jul.-Sept. | | - 0.120 (0.081) | | | | | |  | | | - 0.008 (0.084) |  | 0.149 (0.103) |  |  |
| Piglets born alive (PBA) | | | | | | |  | < 0.01 | | |  | < 0.01 |  | 0.15 |  |
| 16 or more pigs | | | 0.837 (0.111) | | | | |  | | | 0.162 (0.099) |  | -0.079 (0.083) |  |  |
| 12-15 pigs | | 0.474 (0.086) | | | | | |  | | | 0.051 (0.057) |  | -0.029 (0.064) |  |  |
| Stillborn piglets (SB) | | | | | |  | | < 0.01 | | |  | < 0.01 |  | < 0.01 |  |
| 0 | | -0.347 (0.069) | | | | | |  | | | -0.390 (0.079) |  | -0.076 (0.059) |  |  |
| 1 | | -0.182 (0.082) | | | | | |  | | | -0.297 (0.079) |  | -0.089 (0.065) |  |  |
| 2 | | 0.010 (0.055) | | | | | |  | | | -0.253 (0.078) |  | 0.016 (0.069) |  |  |
| Gestation length (GL) | | | | | |  | | < 0.01 | | |  | < 0.01 |  | < 0.01 |  |
| 113-110 days | | 0.522 (0.100) | | | | | |  | | | 0.381 (0.082) |  | 0.151 (0.107) |  |  |
| 114-116 days | | 0.225 (0.070) | | | | | |  | | | 0.038 (0.069) |  | 0.022 (0.078) |  |  |
| Parity x season | |  | | | | | | 0.63 | | |  | 0.42 |  | 0.05 |  |
| Parity x PBA | |  | | | | | | < 0.01 | | |  | 0.04 |  | < 0.01 |  |
| Parity x GL | |  | | | | | | 0.13 | | |  | 0.27 |  | 0.16 |  |
| Parity x SB | |  | | | | | | 0.02 | | |  | 0.03 |  | 0.23 |  |
| Season x PBA | |  | | | | | | 0.20 | | |  | 0.20 |  | 0.05 |  |
| Season x GL | |  | | | | | | 0.98 | | |  | 0.54 |  | 0.37 |  |
| Season x SB | |  | | | | | | 0.33 | | |  | 0.45 |  | 0.08 |  |
| PBA x GL | |  | | | | | | < 0.01 | | |  | 0.28 |  | 0.44 |  |
| PBA x SB | |  | | | | | | < 0.01 | | |  | 0.06 |  | 0.09 |  |
| SB x GL | |  | | | | | | < 0.01 | | |  | 0.05 |  | 0.03 |  |
| Year | |  | | | | | | 0.52 | | |  | < 0.01 |  | 0.04 |  |
| 2015 | -0.046 (0.072) | | | | | | | | |  | -0.175 (0.064) |  | -0.147 (0.069) |  |  |
| Sow herd size | | 0.001 (0.001) | | | | | | 0.39 | | | 0.001 (0.001) | 0.14 | 0.001 (0.001) | 0.10 |  |
| Intercept variance at farm level | | | | 0.75 (0.13) | | | | |  | | 1.72 (0.30) |  | 1.75 (0.32) |  |  |
| Intercept variance at sow level | | | | 1.00 (0.01) | | | | |  | | 0.99 (0.01) |  | 0.96 (0.01) |  |  |
| ICC (records within the same farm), % | | | | | 24.8 | | | - | | | 34.3 | - | 39.2 |  |  |
| ICC (records within the same sow), % | | | | | 7.8 | | | - | | | 45.2 | - | 45.2 |  |  |

^1^SE: standard error; ICC: intraclass correlation coefficient.

^2^Reference categories were sows with BA 12 or less pigs, October-December farrowing, parity 5 or higher, GL 117 days or longer and SB 3 or more pigs.
