# Supplementary material for: Five risk factors and their interactions of probability for a sow in breeding herds having a piglet death during days 0–1, 2–8 and 9–28 days of lactation
Source: Porcine Health Manag. 2021 Aug 30;7:50. doi: 10.1186/s40813-021-00231-0 (PMC8404260; doi:10.1186/s40813-021-00231-0)
Supplement: Supplementary file 2 — Two-way comparisons of pre-weaning piglet mortality risk for sows (probabilities of a sow having a piglet death: PWM) during early (0-1 days), mid- (2-8 days) or late (9-28 days) lactation between parity and piglets born alive (PBA) groups1. [file 40813_2021_231_MOESM2_ESM.docx]

**Additional file 2**. Two-way comparisons of pre-weaning piglet mortality risk for sows (probabilities of a sow having a piglet death: PWM) during early (0-1 days), mid- (2-8 days) or late (9-28 days) lactation between parity and piglets born alive (PBA) groups^1^

| PBA groups | Parity groups | | |
| --- | --- | --- | --- |
|  | 1 | 2-4 | 5 or higher |
|  | Number of sows | | |
| 16 or more piglets | 17,003 | 28,108 | 24,221 |
| 12-15 piglets | 20,057 | 42,402 | 32,912 |
| 11 or less piglets | 18,575 | 52,904 | 28,251 |
|  | Mean (± SE) | | |
|  | PWM during early lactation, % | | |
| 16 or more piglets | 41.6 (1.94)^ay^ | 44.0 (2.06)^axy^ | 46.2 (2.00)^ax^ |
| 12-15 piglets | 33.2 (1.64)^by^ | 35.3 (1.66)^bxy^ | 36.7 (1.73)^bx^ |
| 11 or less piglets | 28.8 (1.47)^c^ | 27.8 (1.63)^c^ | 27.8 (1.69)^c^ |
|  | PWM during mid-lactation, % | | |
| 16 or more piglets | 29.1 (2.81)^ax^ | 29.8 (2.69)^ax^ | 23.9 (2.55)^ay^ |
| 12-15 piglets | 25.0 (2.43)^bx^ | 24.7 (2.28)^bx^ | 21.2 (2.14)^by^ |
| 11 or less piglets | 22.8 (2.51)^bx^ | 22.7 (2.17)^cx^ | 20.0 (1.54)^cy^ |
|  | PWM during late lactation, % | | |
| 16 or more piglets | 15.1 (2.00)^x^ | 13.7 (1.87)^ax^ | 10.1 (1.47)^y^ |
| 12-15 piglets | 14.5 (1.92)^x^ | 12.4 (1.70)^bx^ | 10.3 (1.44)^y^ |
| 11 or less piglets | 14.0 (1.93)^x^ | 12.2 (1.68)^bx^ | 10.6 (1.49)^y^ |

^1^ Means and SEs were estimated in mixed-effects models.

^a-c^Different superscripts within a column represent significant differences in means (P < 0.05).

^x-z^Different superscripts within a row represent significant differences in means (P < 0.05).
