# Supplementary material for: Five risk factors and their interactions of probability for a sow in breeding herds having a piglet death during days 0–1, 2–8 and 9–28 days of lactation
Source: Porcine Health Manag. 2021 Aug 30;7:50. doi: 10.1186/s40813-021-00231-0 (PMC8404260; doi:10.1186/s40813-021-00231-0)
Supplement: Supplementary file 3 — Two-way comparisons of pre-weaning piglet mortality risk for sows (probabilities of a sow having a piglet death: PWM) during early (0-1 days) or mid- (2-8 days) lactation between parity and stillborn piglet groups1. [file 40813_2021_231_MOESM3_ESM.docx]

**Additional file 3**. Two-way comparisons of pre-weaning piglet mortality risk for sows (probabilities of a sow having a piglet death: PWM) during early (0-1 days) or mid- (2-8 days) lactation between parity and stillborn piglet groups^1^

| Stillborn piglet groups (pigs) | Parity groups | | | |
| --- | --- | --- | --- | --- |
|  | 1 | 2-4 | 5 or higher | |
|  | Number of sows | | | |
| 0 | 29,656 | 62,817 | 35,051 | |
| 1 | 13,291 | 30,378 | 21,516 | |
| 2 | 6,918 | 16,978 | 14,598 | |
| 3 or more | 5,770 | 13,141 | 14,219 | |
|  | Mean (± SE) | | | |
|  | PWM during early lactation, % | | | |
| 0 | 30.0 (1.44)^b^ | 32.0 (1.56)^c^ | | 32.6 (1.62)^bx^ |
| 1 | 31.6 (1.63)^by^ | 33.7 (1.69)^cxy^ | | 34.9 (1.74)^bx^ |
| 2 | 36.6 (1.68)^a^ | 36.7 (1.82)^b^ | | 38.3 (1.96)^a^ |
| 3 or more | 39.5 (1.95)^a^ | 39.5 (2.02)^a^ | | 40.1 (1.90)^a^ |
|  | PWM during mid-lactation, % | | | |
| 0 | 23.0 (1.88)^cx^ | 23.6 (2.17)^bx^ | | 19.7 (2.02)^by^ |
| 1 | 23.6 (2.39)^cx^ | 24.7 (2.27)^bx^ | | 20.4 (2.13)^by^ |
| 2 | 26.1 (2.63)^bx^ | 26.1 (2.40)^bx^ | | 21.7 (2.29)^aby^ |
| 3 or more | 30.0 (3.02)^ax^ | 28.4 (2.63)^ax^ | | 23.4 (2.46)^ay^ |

^1^ Means and SEs were estimated in mixed-effects models.

^a-c^Different superscripts within a column represent significant differences in means (P < 0.05).

^x-z^Different superscripts within a row represent significant differences in means (P < 0.05).
