# Supplementary material for: Five risk factors and their interactions of probability for a sow in breeding herds having a piglet death during days 0–1, 2–8 and 9–28 days of lactation
Source: Porcine Health Manag. 2021 Aug 30;7:50. doi: 10.1186/s40813-021-00231-0 (PMC8404260; doi:10.1186/s40813-021-00231-0)
Supplement: Supplementary file 4 — Two-way comparisons of pre-weaning piglet mortality risk for sows (probabilities of a sow having a piglet death: PWM) during early-lactation (0-1 days) between piglets born alive groups and gestation length groups, and between piglets born alive groups and stillborn piglet groups1. [file 40813_2021_231_MOESM4_ESM.docx]

**Additional file 4**. Two-way comparisons of pre-weaning piglet mortality risk for sows (probabilities of a sow having a piglet death: PWM) during early-lactation (0-1 days) between piglets born alive groups and gestation length groups, and between piglets born alive groups and stillborn piglet groups ^1^

| Gestation length or stillborn piglet groups | | Piglets born alive groups | | |
| --- | --- | --- | --- | --- |
|  |  | 16 or more | 12-15 | 11 or less |
| Gestation length groups (days) | | Number of sows | | |
| 110-113 | | 9,537 | 14,723 | 13,617 |
| 114-116 | | 47,521 | 68,837 | 70,981 |
| 117-120 | | 12,174 | 11,811 | 15,132 |
|  | | Mean (± SE) | | |
|  | | PWM during early-lactation, % | | |
| 110-113 | | 47.4 (2.12)^ax^ | 38.2 (1.89)^ay^ | 32.7 (1.96)^ay^ |
| 114-116 | | 44.1 (2.02)^bx^ | 35.0 (1.68)^by^ | 27.4 (1.47)^bz^ |
| 117-120 | | 40.2 (1.88)^cx^ | 32.1 (1.50)^cy^ | 24.3 (1.37)^cy^ |
|  | |  | | |
| Stillborn piglet groups (pigs) | Number of sows | | | |
| 0 | | 33,207 | 46,292 | 48,025 |
| 1 | | 14,854 | 24,140 | 26,191 |
| 2 | | 9,481 | 14,239 | 14,774 |
| 3 or more | | 11,690 | 10,700 | 10710 |
|  | | Mean (± SE) | | |
|  | | PWM during early-lactation, % | | |
| 0 | | 41.2 (1.98)^bx^ | 31.2 (1.52)^cy^ | 23.5 (1.26)^cz^ |
| 1 | | 42.2 (1.90)^bx^ | 32.8 (1.61)^cy^ | 26.1 (1.53)^bz^ |
| 2 | | 45.2 (1.95)^ax^ | 36.6 (1.75)^by^ | 30.3 (1.91)^az^ |
| 3 or more | | 47.2 (2.08)^ax^ | 39.9 (1.87)^ay^ | 32.7 (1.89)^az^ |

^1^ Means and SEs were estimated in mixed-effects models.

^a-d^Different superscripts within a column represent significant differences in means (P < 0.05).

^x-z^Different superscripts within a row represent significant differences in means (P < 0.05).
