# Supplementary material for: Five risk factors and their interactions of probability for a sow in breeding herds having a piglet death during days 0–1, 2–8 and 9–28 days of lactation
Source: Porcine Health Manag. 2021 Aug 30;7:50. doi: 10.1186/s40813-021-00231-0 (PMC8404260; doi:10.1186/s40813-021-00231-0)
Supplement: Supplementary file 5 — Two-way comparisons of pre-weaning piglet mortality risk for sows (probabilities of a sow having a piglet death: PWM) during early (0-1 days) or late (9-28 days) lactation between gestation length and stillborn piglet groups1. [file 40813_2021_231_MOESM5_ESM.docx]

**Additional file 5**. Two-way comparisons of pre-weaning piglet mortality risk for sows (probabilities of a sow having a piglet death: PWM) during early (0-1 days) or late (9-28 days) lactation between gestation length and stillborn piglet groups^1^

| Stillborn piglet groups (pigs) | Gestation length groups (days) | | |
| --- | --- | --- | --- |
|  | 110-113 | 114-116 | 117-120 |
|  | Number of sows | | |
| 0 | 16,731 | 92,088 | 18,705 |
| 1 | 9,328 | 46,555 | 9,302 |
| 2 | 5,967 | 26,732 | 5,795 |
| 3 or more | 5,851 | 21,964 | 5,315 |
|  | Mean (± SE) | | |
|  | PWM during early lactation, % | | |
| 0 | 34.2 (1.68)^dx^ | 31.3 (1.54)^dy^ | 29.1 (1.41)^cz^ |
| 1 | 37.1 (1.83)^cx^ | 33.3 (1.70)^cy^ | 30.0 (1.56)^cz^ |
| 2 | 40.8 (2.17)^bx^ | 36.9 (1.72)^by^ | 33.9 (1.60)^bz^ |
| 3 or more | 45.4 (2.34)^ax^ | 39.6 (1.89)^ay^ | 34.5 (1.75)^az^ |
|  | PWM during late lactation, % | | |
| 0 | 13.2 (1.80)^x^ | 11.6 (1.54)^bxy^ | 11.3 (1.49)^by^ |
| 1 | 12.4 (1.72) | 11.8 (1.58)^ab^ | 11.7 (1.59)^b^ |
| 2 | 14.5 (1.98)^x^ | 12.1 (1.64)^aby^ | 12.3 (1.60)^ay^ |
| 3 or more | 13.9 (1.86) | 12.6 (1.72)^a^ | 12.3 (1.71)^a^ |

^1^ Means and SEs were estimated in mixed-effects models.

^a-d^Different superscripts within a column represent significant differences in means (P < 0.05).

^x-z^Different superscripts within a row represent significant differences in means (P < 0.05).
