# Supplementary material for: Five risk factors and their interactions of probability for a sow in breeding herds having a piglet death during days 0–1, 2–8 and 9–28 days of lactation
Source: Porcine Health Manag. 2021 Aug 30;7:50. doi: 10.1186/s40813-021-00231-0 (PMC8404260; doi:10.1186/s40813-021-00231-0)
Supplement: Supplementary file 6 — Spearman correlation matrix for the number of piglet deaths during early (0-1 days), mid- (2-8 days) or late (9-28 days) lactation. [file 40813_2021_231_MOESM6_ESM.docx]

**Additional file 6.** Spearman correlation matrix for the number of piglet deaths during early (0-1 days), mid- (2-8 days) or late (9-28 days) lactation

|  |  | Piglet deaths |  |
| --- | --- | --- | --- |
|  | Early lactation | Mid-lactation | Late lactation |
| Mid-lactation | 0.02* | 1.00 | - |
| Late lactation | 0.02* | 0.12* | 1.00 |

*indicates *P* < 0.01.
